# Episodic memory differences in social and non-social contexts

**DOI:** 10.1371/journal.pone.0342919

**Published:** 2026-04-02

**Authors:** Karina Grunewald, Susanne Schweizer

**Affiliations:** School of Psychology, University of New South Wales, Sydney, Australia; Nanjing University, CHINA

## Abstract

Humans use existing knowledge to predict and recall events. As a social species, it has been argued that humans preferentially process and recall social information. Individuals then should be better at using prior knowledge of actors’ character traits to predict and remember their behaviour in social contexts compared to predicting and remembering trait-consequence associations in non-social contexts. To test this hypothesised social episodic memory advantage, we modified a social episodic memory paradigm to include a social and non-social condition. 215 participants (18–65 years; 43% female) learnt social (people) and non-social (airports) actors’ traits, predicting and remembering their subsequent actions across various fictional events. Participants showed better memory for social compared to non-social events. However, only in non-social contexts was recall aided by prior knowledge of the actors (i.e., recall was better for events that were consistent with previously learnt information). Participants also showed a positivity bias for both social (e.g., kind actions by others) and non-social (e.g., flights running efficiently) information recall. Social memory then is preferentially processed, and social information recalled better, regardless of whether it fits with individuals’ prior knowledge or not. This may be particularly the case in situations where information about social actors is limited and all information is critical to inform whether an individual is safe to affiliate with or should be avoided. The findings also provide preliminary evidence that positive information may be preferentially recalled in these contexts. Together these findings support better memory for social over non-social information.

## Introduction

Throughout our lives, we must learn to interact with and navigate the complex social environments that surround us. Episodic memory, here defined as the ability to register and retrieve specific details associated with past experiences [1,2], allows us to recall the past to make predictions about future events [3–5]. This ability is critical to functioning across a range of domains, including planning, decision-making, and problem solving [6,7]. In social contexts, episodic memory allows us to learn about individuals’ traits and use this existing knowledge to understand and predict people’s behaviours [8]. Much work has explored social episodic memory. However, while social memory has been proposed to be preferentially processed over non-social memory, this propagated social memory advantage remains little explored. The current study aimed to bridge this gap by examining whether and how episodic memory differs in social compared to non-social contexts.

The dynamic nature of our species’ large, complexly bonded social groups means humans continuously encounter novel social situations and actors [9], and the human neocortex has been proposed to have evolved to process information associated with the dynamic groups that make up human societies [10]. Social episodic memory helps guide individuals’ behaviour in novel social situations. The schema theory proposes that humans form schemas based on prior information about social targets. These schemas are activated when processing new information about said targets [11,12]. Information that is consistent with existing schemas is better remembered than inconsistent information [3]. For example, episodic memory for social information is more accurate when it is consistent with previous knowledge of social targets’ traits and behaviours compared to memory for inconsistent information [2,3,13]. This knowledge can then be used to make predictions about social targets’ future behaviours [8]. This allows individuals to maximise payoff in social interactions, as social episodic memory can inform their decisions on who to approach or avoid in novel social situations [13]. Episodic memory may thus be more accurate when it is consistent with prior knowledge. If a social memory advantage is indeed observed in episodic memory, then this accuracy may be further potentiated in social compared to non-social contexts, and individual differences in episodic memory may also be predictive of social functioning.

The social information processed in memory is inherently valenced (e.g., friend or foe; acceptance or rejection), and studies have found that episodic memory recall is better for valenced compared to neutral information [14,15], especially for negative social information, experiences and associations [16–18]. A negativity bias in memory has been proposed to be generally adaptive, as it sensitises individuals to potential environmental risks [19]. However, in social situations, adopting a negativity bias may limit opportunities for social connection, thus potentially having more adverse impacts in social compared to non-social contexts. Negativity biases in memory have also been linked to increased risk of maladaptive cognitive processes, such as rumination and sensitivity to social rejection, and even increased symptoms of mental health disorders [20–23]. Interestingly, while many studies have traditionally focused on negativity biases in memory, researchers have also observed positivity biases (i.e., increased recall for positive events and associations) in memory (for a review, see: 15). It is therefore important to investigate how episodic memory may be differentially affected by negative and positive information. As with consistency effects, any observed valence effects may also be greater under social compared to non-social contexts given a social memory advantage.

Here, we modified an existing social episodic memory paradigm [2] to examine whether episodic memory varied for predictions of social vs non-social events. As previous studies have typically focused on investigating the effects of variables such as consistency and valence on episodic memory within social contexts only, we aimed to introduce a non-social condition to existing paradigms to directly compare episodic memory differences within social and non-social contexts. In the task, participants learnt about social (people) and non-social (airports) targets’ traits and then made predictions about positive and negative events including these targets. Half the events were consistent and the other half inconsistent with participants’ prior knowledge of the targets. The task design allowed us to investigate the effects of consistency and valence. Specifically, we predicted (https://osf.io/63jb7) that episodic memory would be more accurate: for social compared to non-social events (H1); for events that are consistent with prior knowledge compared to those that are inconsistent (H2a), especially in social contexts (H2b); and for negative compared to positive events (H3a), especially in social contexts (H3b). Finally, we hypothesised that better social memory would be associated with indices of real-world social functioning (incl. size, quality, and self-rated satisfaction with participants’ social network; H4).

## Methods

This study was approved by the University of New South Wales Human Research Executive Committee (HC Number 3800).

### Participants

Participants were recruited through Prolific until 230 unique individuals completed the study faithfully (i.e., failed no more than 2 out of 5 attention check items included throughout the experiment), as per the pre-registration. Power simulations using the ‘mixedpower’ package [24] were run across various sample sizes (50–400) to compute a sample size calculation testing for significance, bootstrapped over 1000 iterations [25]. Condition, consistency, and valence were added as constants based on the expected directions of their main effects, with accuracy (% correct) added as the dependent variable. A sample size of approximately 230 was estimated to detect a main effect of all three constants at a power of 0.80. As two of the recruited participants failed 3 attention check items, these participants were excluded and two more were recruited in their place.

To participate in the study, individuals had to be: aged 18–65 years; fluent in English, as the study task and assessments relied on adequate reading ability; and have no history of head injury, neurological disorder, or diagnosed learning disabilities, as the task placed significant demands on cognitive abilities (i.e., episodic memory), which are often impaired in these individuals. To ensure participants met inclusion criteria prior to commencing the study, screening was conducted at the recruitment stage using Prolific’s existing pre-screening filters. Participants who met these criteria and provided informed consent were awarded £9 through Prolific upon study completion (as per the platform’s guidelines for fair and equitable compensation). To incentivise participation, participants were additionally awarded an extra £1 if they scored above 80% correct across all conditions of the Episodic Memory Task, and £3 if they scored above 95% across all conditions.

Fifteen participants were additionally excluded for performing at or below chance on the Episodic Memory Task (responding accurately on 50% or less of the trials). The final sample comprised 215 participants (Table 1).

**Table 1:** Sample characteristics (N = 215).

	*Mean (SD)/N (%)*
**Age (years)**	38.48 (11.04)
**Gender**	
Female	93 (43.26%)
Male	120 (55.81%)
Non-binary	1 (0.47%)
Prefer not to say	1 (0.47%)
**Ethnicity**	
Black	23 (10.70%)
Asian	33 (15.35%)
White	143 (66.51%)
Hispanic	6 (2.79%)
Mixed	7 (3.26%)
Prefer not to say	3 (1.40%)
**Highest Education**	
Primary School	2 (0.93%)
High School	41 (19.07%)
Training	24 (11.16%)
University	148 (68.84%)
**Wealth**	
Not at all	15 (6.98%)
Not very	67 (31.16%)
Fairly	115 (53.49%)
Rather	17 (7.91%)
Very	1 (0.47%)
**Depressive symptoms**	
None	111 (51.63%)
Mild	54 (25.12%)
High	41 (19.07%)
Missing data	9 (4.19%)
**Social sensitivity**	20.06 (11.23)
**Size**	10.71 (10.48)
In person	4.36 (4.24)
Online	6.36 (7.33)
**Quality**	7.90 (7.66)
Favour	4.51 (4.78)
Secret	3.38 (3.45)
**Satisfaction**	139.62 (42.64)
In person	66.14 (28.20)
Online	73.47 (19.73)
**Support**	68.01 (24.80)

Training = Professional/Vocational Training; Wealth = how wealthy participants think they are; Depressive symptoms = levels of depressive symptoms on the Patient Health Questionnaire [26] (none = 0–4; mild = 5–9; high = 10+); Social sensitivity = total score on the Online and Offline Social Sensitivity Scale [27]; Size = sum of total number of in person and online friends; Quality = total number of friends participants could ask a favour of and would trust to keep a secret; Satisfaction = sum of reported happiness with how often participants spend time with friends in-person (on a scale from 1–100) and online (on a scale from 1–100); Support = how supported participants feel by their friends (on a scale from 1–100).

### Procedure

After providing informed consent, participants completed questionnaires assessing demographics, depressive symptoms, social sensitivity, and real-world social networks, followed by the episodic memory task (S1 Fig). Altogether, the study took approximately one hour to complete.

#### Questionnaires.

Participants first completed questions about their demographics, including age, gender, ethnicity, socioeconomic status (using highest degree of education as proxy), and perceived socioeconomic status.

Depression was measured with the Patient Health Questionnaire (PHQ; 26). The questionnaire is made up of 8 items assessing severity of symptoms of depression over the last two weeks on a 4-point Likert scale. The 9^th^ item, measuring suicidality, was excluded from the current study, as risk could not be managed in the context of online data collection. Items are rated from 0 (*not at all*) to 3 (*nearly every day*). The scale demonstrated good internal consistency in the current sample (ω_T_ = .92). Scores on the PHQ items were added and depressive symptoms were indexed as a three-level variable: none (scores of 0–4), mild (scores of 5–9) and high (scores of 10 and above). These scores differentiate between individuals with no significant depressive symptoms, mild levels of depressive symptoms, and moderate and severe levels of depressive symptoms respectively [26].

Social sensitivity was assessed with the Online and Offline Social Sensitivity Scale (O^2^S^3^; Andrews, Khin, et al., 2021). The scale is made up of 18 items assessing social sensitivity in an online and offline context. Items are rated on a 4-point Likert scale ranging from 0 (*strongly disagree*) to 3 (*strongly agree*). The scale demonstrated good internal consistency (ω_T_ = .95). Social sensitivity was indexed as the total score on the O^2^S^3^, with higher scores indicating higher social sensitivity.

Participants then completed custom questions assessing the size, quality, and satisfaction with their real-world social networks. *Social network size* was assessed as the number of friends participants regularly interact with in person and online. For online networks, participants also reported the number of people they “followed” and that were “following” them on their most used social media platform. *Social network quality* was measured as the number of friends participants could ask a favour of and would trust to keep a secret. *Social network satisfaction* was assessed as participants’ self-rated satisfaction with how often they interact with friends online and offline, and how supported they feel by their friends, on scales ranging from 0–100.

#### Episodic memory task.

After completing all questionnaires, participants then began the Episodic Memory Task, adapted from Udeogu et al. (2022). The task comprised two conditions presented in counterbalanced order: a non-social condition in which participants learnt about airports and their flight connections and a social condition in which participants learnt about people and their social connections. Each condition was made up of six phases. Once a phase was completed, participants immediately began the next phase, with self-paced instructions provided at the beginning of each phase before the first trial began.

**Fig 1:**
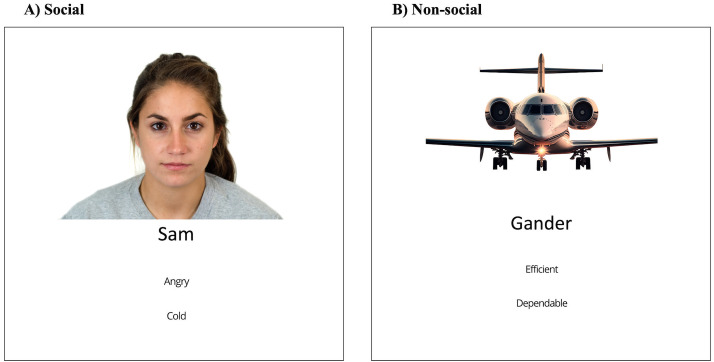
Episodic Memory Task: Phase 1. A schematic overview of phase 1, where participants learn target-trait attributes. **(A)** Social condition. **(B)** Non-social condition.

In phase 1, participants first learnt trait information about twelve different airports (non-social) or people (social). In the non-social condition, six airports had traits indicative of being organised (e.g., efficient), and six airports had traits indicative of being disorganised (e.g., slow). In the social condition, six people had traits indicative of being nice (e.g., caring), and six people had traits indicative of being mean (e.g., cruel). Participants were shown these trait-airport/person associations twice, each time in a different pseudorandom order, so that the first and last trials were made up of an organised/nice airport/person, with each individual association shown for 6 seconds (Fig 1). Participants thus formed trait-target associations.

**Fig 2:**
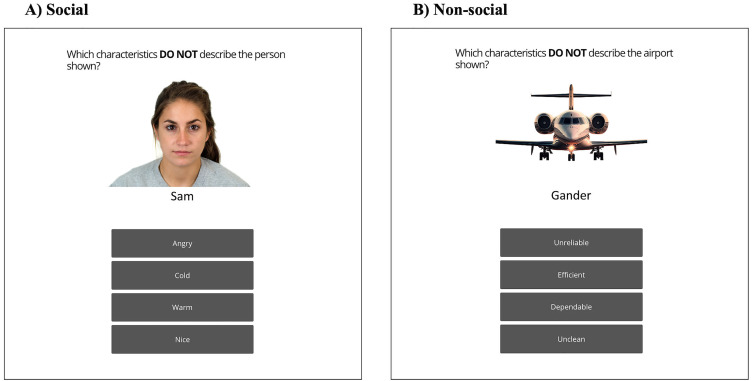
Episodic Memory Task: Phase 2. A schematic overview of phase 2, where participants are asked to identify which attributes are not associated with the given targets. **(A)** Social condition. **(B)** Non-social condition.

In phase 2, to test whether participants had indeed learnt the trait-target associations during the learning phase, they were then presented with the same trait-airport/person associations as phase 1 (in a different pseudorandom order), along with two additional attributes of the opposite trait. Participants were asked to identify which of the four attributes did not describe the airport/person shown. For example, a person that was originally associated with “mean” attributes was then shown with the two traits they were initially presented with (e.g., angry, cold), along with two nice traits (e.g., thoughtful, sweet). Participants had to identify the incorrect attributes within 7 seconds in each trial (Fig 2; S1 Table).

**Fig 3:**
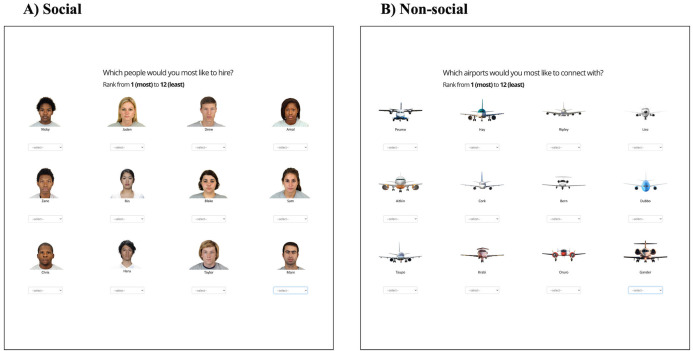
Episodic Memory Task: Phase 3. A schematic overview of phase 3, where participants rank targets in the order they would like to interact with them. **(A)** Social condition. **(B)** Non-social condition.

In phase 3, participants were then given 2 minutes to rank the airports/people in the order they would like to establish flight routes with them if they were a transport manager (non-social) or would like to hire them if they were a hiring manager (social; Fig 3). This initial ranking was done as a secondary check to test whether participants had learnt the trait-target associations, as well as a check to see how these learnt associations may impact participant approach/avoidance behaviours to the targets.

**Fig 4:**
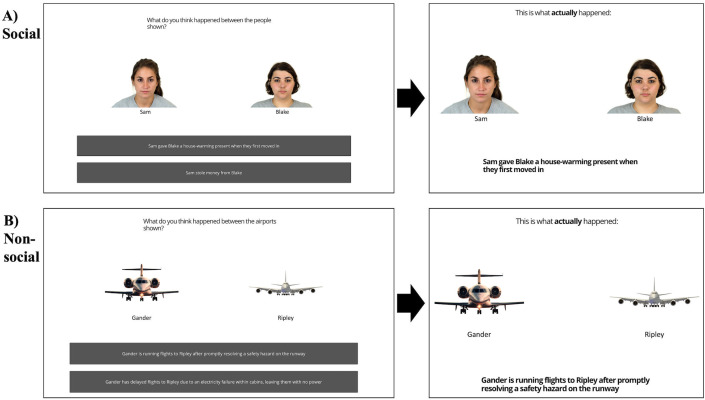
Episodic Memory Task: Phase 4. A schematic overview of phase 4, where participants predict outcomes based on learnt target-trait associations and are shown which outcomes actually took place. **(A)** Social condition. **(B)** Non-social condition.

In phase 4, participants read scenarios involving two airports/people they previously learnt about. They were shown two possible actions that target A may have engaged in in relation to target B (one positive and one negative; see S1 Appendix for a full list of behavioural sentences used in the task). Participants had 15 seconds to select which of the two actions they believed actually took place (i.e., predicting which action they believed target A engaged in) based on the target-trait associations they had previously learnt. After making a prediction, they were immediately shown the event that actually took place between the airports/people shown. The correct outcomes were shown for 5.5 seconds (Fig 4). Predictions were scored based on whether they were consistent or inconsistent with the learnt trait associations about target A. It is important to note that while it is possible that participants could encode the actions that took place based on their knowledge of target B, rather than target A, any effects that this might have on observed results were mitigated by including one negative and one positive action for target A.

This phase introduced the scenarios participants would then be asked to recall in the following phase. It also functioned as an additional check of whether participants had indeed learnt the target-trait associations, and to what extent they might then rely on these learnt associations when making predictions about the targets’ behaviours.

In total, there were 48 scenarios per condition. Each of the twelve airports/people were shown as the actor (i.e., target A) in four scenarios: two with outcomes consistent with the learnt trait associations (e.g., a nice person doing a positive action); two with outcomes inconsistent with the learnt trait associations (e.g., a disorganised airport with flights running); two (one consistent and one inconsistent) with positive outcomes (e.g., “A is running flights to B due to good management”) and two (one consistent and one inconsistent) with negative outcomes (e.g., “A refused to let B join them for lunch”).

Trials were presented in a pseudorandom order, so that no more than four consistent/inconsistent, positive/negative, and male/female (in the social condition) trials were presented consecutively, no same airport/person was presented as the actor (i.e., target A) on consecutive trials, and the first and last trials were made up of positive outcomes (S1 Table).

**Fig 5:**
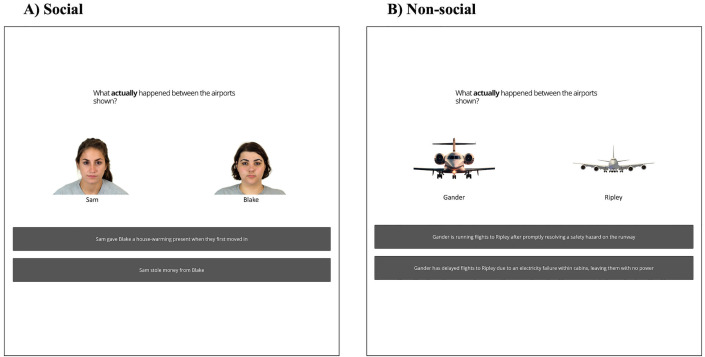
Episodic Memory Task. A schematic overview of phase 5, where participants are asked to recall which outcomes took place. **(A)** Social condition. **(B)** Non-social condition.

In phase 5, participants were then asked to recall which outcomes actually took place between the airports/people shown in phase 4. They had 10 seconds to answer each trial, with trials presented in a pseudorandom order as described for phase 4 (Fig 5). This was the main experimental manipulation, with task accuracy operationalised as the total number of outcomes that were correctly recalled in this phase in each condition (S1 Table). Consistency was operationalised as whether the outcomes being recalled were consistent or inconsistent with the previously learnt target-trait associations, and valence was operationalised as whether the outcome being recalled was positive (i.e., a positive action being performed) or negative (i.e., a negative action being performed).

Lastly, in phase 6, participants were given another 2 minutes to rank the airports/people in the order that they would like to establish flight routes with them if they were a transport manager (non-social) or would like to hire them if they were a hiring manager (social; Fig 3). This post-task ranking was done to check whether the original approach/avoidance behaviours from the initial ranking would change, and if so how, once participants learnt more about actions taken by the targets.

The images in the non-social condition were made up of airplanes (representing airports) licensed from Adobe Stock (https://stock.adobe.com/), over a white background. In the social condition, the images were made up of neutral faces from the Chicago Face Database [28] and the London Face Research Set [29] over a white background. Person and airport traits were derived from an existing database of English lemmas [30], controlling for valence of positive and negative traits across the two conditions. A t-test indicated no significant difference in valence for the final list of trait adjectives between the two conditions (positive traits: *t* = 2.06, *df* = 22. *p* = .057; negative traits: *t* = 1.85, *df* = 22. *p* = .078). Where possible, the behavioural sentences used in the social condition were modified from an existing list of 400 behavioural statements [31] that have been used in similar studies. For a complete list of the traits and behavioural statements used, see S1 Appendix. For descriptive statistics of episodic memory task performance, see S1 Table.

### Analyses

Reported statistics were based on linear mixed models including participant ID as a random effect, unless otherwise specified. Effect sizes were calculated using r squared, as has been recommended for mixed effects models [32].

To assess whether participants learnt the person/airport trait associations, preliminary analyses were conducted with condition (non-social vs social) entered as a fixed effect and phase 2 accuracy (operationalised as correctly identified target-trait associations) entered as outcome. To investigate whether participants used the learnt trait associations to predict behaviours in phase 4, condition (non-social vs social) was entered as a fixed effect and phase 4 prediction accuracy (operationalised as total number of predictions in phase 4 that were consistent with target-trait associations) entered as outcome.

Hypothesis 1 was tested with condition (non-social vs social) entered as a fixed effect and phase 5 accuracy (operationalised as number of total trials correctly recalled) entered as outcome. Exploratory analyses were run with phase 2 accuracy added as a fixed effect to the model testing H1, to investigate whether any observed effects of condition would remain significant while controlling for differential recall of target-trait associations. Hypothesis 2a was tested with consistency of behaviour with prior trait knowledge entered as a fixed effect (consistent vs inconsistent), and phase 5 accuracy entered as outcome. To answer hypothesis 2b, condition (non-social vs social) was added as a fixed effect to the model testing H2a. Hypothesis 3a was tested with valence of behaviour (negative vs positive) entered as a fixed effect, and phase 5 accuracy entered as outcome. To answer hypothesis 3b, condition (non-social vs social) was added as a fixed effect to the model testing H3a. Hypothesis 4 was tested with social network size (total number of friends regularly seen in person and online), quality (total number of friends trusted to keep a secret and do a favour), satisfaction (self-rated satisfaction with how often participants see friends in person and online) and support (how supported participants felt by their friends) separately added as fixed effects into the model testing H1.

For pre-registered exploratory analyses, depression and social sensitivity were separately added as fixed effects to the models testing H1 and H3, as these factors are associated with heightened negative memory bias [22,23]. As most of these exploratory analyses were not significant or had very small effects (supplementary materials, S11-S12 Tables) they are therefore not discussed further.

### Transparency and openness

We report our sample size calculation, participants inclusion requirements, participant exclusions, manipulations and measures in the study within the manuscript. The study follows JARS [33]. On publication of the manuscript, de-identified data and code will be made available on the project page on the Open Science Framework (https://osf.io/63jb7). Study materials will not be made available, as some of the included images are licensed to the authors. This study’s design and analyses were pre-registered prospectively, prior to participant recruitment and data collection (https://osf.io/63jb7).

All statistical analyses were conducted using R version 4.2.1 [34]. Linear mixed models were conducted using the lme4 package [35], and significant effects were further investigated with contrasts using the lsmeans package [36]. Correlations were conducted using the apaTables package [37]. Effect sizes were calculated using the MuMIn package [38].

## Results

### Preliminary analyses

Preliminary analyses showed that participants learnt the person/airport trait associations and then used these to predict the behaviours of the people/airports in novel situations. Recall was better for social (72.56%, *SD* = 26.88) compared to non-social target-trait associations (60.91%, *SD* = 25.28) in phase 2 (*F* = 37.90, *df* = 214.00, *p* < .001, *R*^*2*^*m* = 0.05, *R*^*2*^*c* = 0.46). Predictions of behaviours in phase 4 were more consistent with previously learnt target-trait associations (i.e., participants predicted positive behaviours for targets with a learnt positive trait association, and negative behaviours for targets with a learnt negative trait association) in the social (71.11%, SD = 16.60) compared to the non-social (58.61%, SD = 13.10) condition (*F* = 127.10, *df* = 214.00, *p* < .001, *R*^*2*^*m* = 0.15, *R*^*2*^*c* = 0.50).

Further supporting evidence of learning trait-target associations, initial trait-based ranking of targets showed that participants ranked targets with positive traits higher than those with negative traits (non-social: *F* = 832.16, *df* = 196.53, *p* < .001, *R*^*2*^*m* = 0.68, *R*^*2*^*c* = 0.68; social: *F* = 1478.80, *df* = 205.53, *p* < .001, *R*^*2*^*m* = 0.79, *R*^*2*^*c* = 0.79). However, no significant differences were observed between initial and post-task ranking of targets (*p* > .05).

In response to reviewer comments, we ran additional exploratory analyses investigating the effects of episodic memory accuracy and valence of target traits (positive vs negative) on differences in ranking of targets from baseline to post task. While no significant differences were observed on ranking (post task minus baseline) as a function of accuracy (*p* > .05), there was a significant effect of target traits’ valence (non-social: *F* = 33.46, *df* = 10.00, *p* < .001, *R*^*2*^*m* = 0.02, *R*^*2*^*c* = 0.02; social: *F* = 22.70, *df* = 10.00, *p* = .001, *R*^*2*^*m* = 0.05, *R*^*2*^*c* = 0.08, S2 Fig).

### Social versus non-social episodic memory

**Fig 6:**
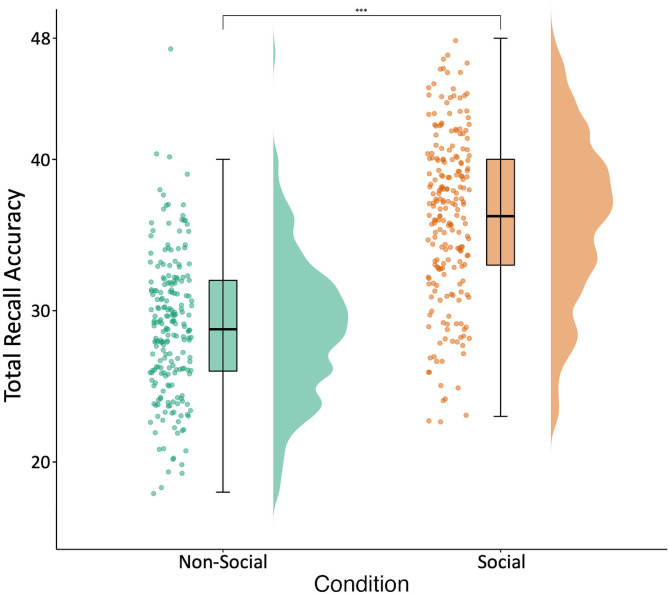
Social and non-social episodic memory accuracy. Accuracy is operationalised as number of total correct trials out of 48 trials. Fig 6 depicts participant accuracy in trials of the test phase of the episodic memory task (phase 5) across the different conditions (non-social vs social). From left to right for each condition, the picture depicts individual data points showing each participants’ total accuracy score (with jittering used to improve data point visibility), followed by a boxplot with the mean accuracy across participants indicated by the bar, and lastly a frequency distribution of participants’ total accuracy scores.

As predicted (H1), episodic memory was significantly more accurate for events in the social compared to the non-social condition (*F* = 376.23, *df* = 214.00, *p* < .001, *R*^*2*^*m* = 0.36, *R*^*2*^*c* = 0.59; Fig 6).

### The effect of consistency varies across social and non-social contexts

**Fig 7:**
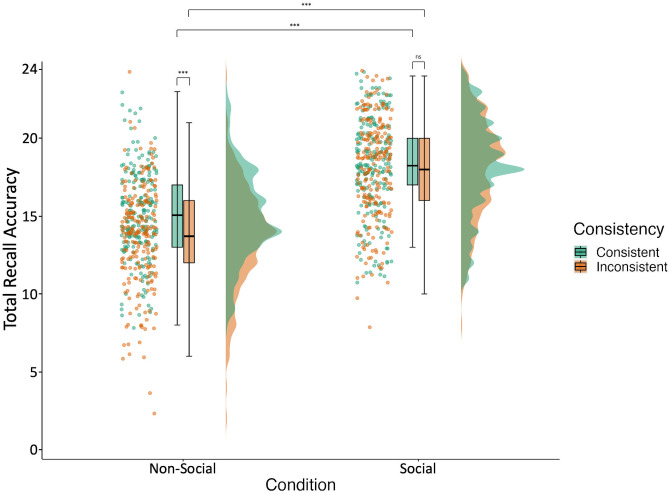
Episodic memory accuracy depending on condition and consistency. Accuracy is operationalised as number of total correct trials (24 consistent; 24 inconsistent). Fig 7 depicts participant accuracy in consistent vs inconsistent trials of the test phase of the episodic memory task (phase 5) across the different conditions (non-social vs social). From left to right for each condition for the different consistency types (consistent vs inconsistent), the picture depicts individual data points showing each participants’ total accuracy score (with jittering used to improve data point visibility), followed by a boxplot with the mean accuracy across participants indicated by the bar, and lastly a frequency distribution of participants’ total accuracy scores.

When investigating the effects of consistency on recall, as predicted (H2a), episodic memory was significantly more accurate for events that were consistent with prior knowledge compared to those that were inconsistent (*F* = 11.63, *df* = 644.00, *p* < .001, *R*^*2*^*m* = 0.01, *R*^*2*^*c* = 0.11). Contrary to H2b, however, while there was a significant interaction between condition and consistency (*F* = 9.28, *df* = 642.00, *p* = .002, *R*^*2*^*m* = 0.28, *R*^*2*^*c* = 0.47; S3 Table), deconstructing this interaction showed a significant effect of consistency in the non-social condition, but not the social condition (Fig 7, S4–S5 Tables).

### Exploring the effects of trait memory recall on sociality and consistency effects

Given the observed differences in memory recall for social compared to non-social target-trait associations, we tested whether the observed condition (social vs. non-social) and consistency (consistent vs. inconsistent) effects were simply an artefact of better trait memory recall in the social condition. Number of accurately recalled target-trait associations was therefore added to the models testing H1-2. The effects of condition and consistency remained significant (S6 Table).

### Better memory for positive compared to negative information

**Fig 8:**
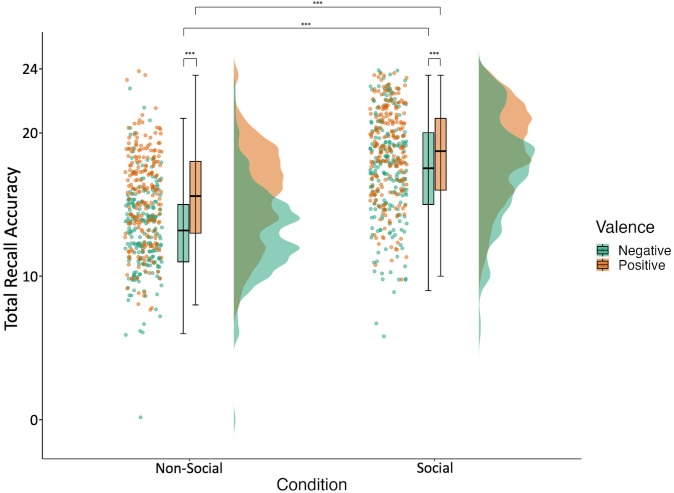
Episodic memory accuracy depending on condition and valence. Accuracy is operationalised as number of total correct trials (24 negative; 24 positive). Fig 8 depicts participant accuracy in negative and positive trials of the test phase of the episodic memory task (phase 5) across the different conditions (non-social vs social). From left to right for each condition for the different valence types (negative vs positive), the picture depicts individual data points showing each participants’ total accuracy score (with jittering used to improve data point visibility), followed by a boxplot with the mean accuracy across participants indicated by the bar, and lastly a frequency distribution of participants’ total accuracy scores.

The valence effect (Fig 8) was in contrast with our prediction (H3a), with memory recall being more accurate for positive compared to negative events (*F* = 51.79, *df* = 644.00, *p* < .001, *R*^*2*^*m* = 0.05, *R*^*2*^*c* = 0.12). A significant valence by condition interaction (Hb3; *F* = 8.97, *df* = 642.00, *p* = .003, *R*^*2*^*m* = 0.29, *R*^*2*^*c* = 0.43; S7 Table) suggested a differential effect of valence in both conditions. Deconstructing the interaction revealed a significant positivity effect in both the social and non-social conditions, but the effect was larger in the non-social condition accounting for 13% of variance in memory performance compared to 3% in the social condition (S8–S9 Tables).

### Episodic memory is unrelated to real-world social networks

Contrary to our predictions, size, quality, self-rated satisfaction and support from participants’ social networks were unrelated to social episodic memory (H4; S10 Table).

## Discussion

Successful navigation of our social environments has lasting impacts on our health and well-being [39–42]. Much of our cognitive capacity, therefore, is recruited in service of social information processing. However, while social episodic memory has been proposed to be preferentially processed, it has not been systematically compared to non-social episodic memory. In this study, we showed that participants recalled social information better compared to non-social information. This finding adds to and is consistent with the existing literature suggesting that social information appears to be preferentially processed and recalled compared to non-social information across different domains of cognition [43–45].

Consistent with this preferential processing of social information, social traits were also learnt better during the learning phase. It is therefore possible that the observed preferential recall of social compared to non-social events could merely be an effect of better learning of social compared to non-social information. However, exploratory analyses suggest this is likely not the only factor influencing the observed effects, as participants showed significantly greater episodic memory recall for social compared to non-social information even after we controlled for differential recall of target-trait associations. Additionally, the differential impacts of consistency and valence suggest that social information is indeed processed differently from non-social information in episodic memory.

There was a main effect of consistency, where, in line with the schema theory [3], consistent information was recalled better. This effect was qualified by an interaction with context, where the effect of consistency was significant in the non-social, but not the social condition. This is in contrast with previous findings, which have shown a consistency effect for social information [2,3,13]. While overall recall in the present study was relatively low, social information recall was significantly higher than non-social recall, with participants displaying on average 76% accurate recall in the social condition (compared to 60% overall accurate recall in the non-social condition). The lack of observed consistency effects in the social condition then could be due to this discrepancy, as participant memory within the social condition was much higher than in the non-social condition, and therefore perhaps no differences in consistency could be observed. This is somewhat supported by the observed valence effects discussed below, as these also showed a greater magnitude in the non-social compared to the social condition.

Alternatively, from a social functioning perspective, inconsistent social information may arguably be as important if not more important than consistent social information to encode when first learning about others. In previous studies [2,3,13], participants often learnt target-trait associations by reading multiple sentences describing behaviours performed by the targets, thus providing contextually enriched social information prior to episodic memory testing. In contrast, the present study only provided trait-adjectives as information, so participants had no prior behavioural knowledge of targets. In contextually impoverished environments, remembering both consistent and inconsistent information about novel social targets then may be critical to better understand and predict these social targets’ characters and behaviours, thus reducing the consistency benefit in the social condition. An alternative account is novelty, as memory for novel information is superior [46], and individuals display overall greater attention to social compared to non-social information [47,48]. Inconsistent social events (e.g., a nice person behaving antisocially) may then carry greater salience relative to inconsistent non-social events (e.g., an organised airport having delays). In the social condition, the consistency benefit then would similarly be comparatively reduced. As this was the first study to use the existing task, future studies should investigate whether the observed effects can be replicated, and, if so, investigate whether any of these proposed differences might be driving them.

In addition to consistency, memory also varied as a function of valence. However, contrary to our hypothesis and previous research showing negative biases across domains of cognition, including learning, decision-making, and working memory [49,50], positive events were better remembered. The observed positivity bias is in line with Levy et al. [51], who similarly found that participants displayed better memory recall for prosocial behaviours compared to non-prosocial behaviours. This preference for encoding prosocial information may confer advantages to individuals in social contexts, as it can inform their decisions on who to approach in novel situations. Indeed, researchers have proposed that positive biases in memory processing may bear advantages [52], as individuals display a tendency to search for positive experiences and avoid negative experiences [53]. The prosociality literature, however, does not speak to the positivity bias in memory for non-social information, which was larger than the social memory advantage. A review of the literature on valence in episodic memory suggests that broad gist memory is better for positive events, whereas event detail is better recalled for negative events [15]. The paradigm in the current study relied on recognition memory rather than detailed recall, which likely accounts for the overall positivity bias observed.

Lastly, while we argued that a social episodic memory advantage benefits social functioning, there was no significant association between real-world network characteristics, such as size and quality, and social episodic memory recall in the present study. Arguably, the lack of self-relevance in the task may obscure associations between a social episodic memory advantage and social functioning as measured by social network and size. Future studies could explore this further, employing connections between real-world social targets that participants are familiar with in the task. Studies have also found that social contexts and interactions themselves may impact working memory performance [54], with researchers proposing a developmental two-way interaction between social experiences and neural development [55]. This potential association between social interactions and working memory performance could also be investigated in future research. Paradigms such as those employed by Mahmoodi et al., [56] could be applied to investigate how participants make predictions about social outcomes, and which memory systems may drive these effects.

It is important to note that the results observed in the present study could be driven by a familiarity effect of social over non-social information. As individuals encounter and interact with other humans daily, they may have greater expertise encoding faces, traits, and behaviours of other humans compared to those of airports and their connections. This could then result in improved recall of more familiar (i.e., social) traits and behaviours. Future studies could explore this by investigating whether the same social memory advantage is observed among individuals that are equally familiar with people and airports (e.g., pilots, airline staff). Alternatively, other non-social conditions could be designed with stimuli that may, on average, be more familiar to individuals (e.g., food, clothes). Importantly, airports and other transport routes may also be processed as social to some extent, as humans are typically involved in the operations of these environments. Including alternative non-social conditions with targets such as food and clothes could then serve to further validate the findings from the present study with more neutral non-social stimuli. Future studies could also populate the paradigm with less distinctive stimuli, such as silhouettes of people or airplanes, to ensure that there are no confounding effects of stimuli’s salience on observed memory effects.

Lastly, it should also be noted that while the present episodic memory task consists of a similar paradigm to existing studies investigating episodic memory processes [2,3,13], due to the nature and simplicity of the stimuli used in the present task, recall of the current stimuli may rely on semantic memory, rather than episodic memory. Future studies should investigate this distinction by including more diverse and immersive stimuli, such as video representations of scenarios that allow participants to experience the context surrounding the events they are learning about, to investigate whether the same effects can be observed in these more immersive conditions. Interestingly, the lack of observed differences between initial and post-task rankings of which targets participants would most like to hire or connect with could suggest that traits may be more important for judgments of targets than behavioural outcomes. Indeed, participants appeared to rank targets with positive traits (i.e., nice people or organised airports) higher at the post-task compared to the baseline ranking. In contrast, they appeared to rank targets with negative traits (i.e., mean people or disorganised airports) lower at the post-task compared to the baseline ranking. While these findings were exploratory, they may provide preliminary support for the importance of traits in both social and non-social judgments. Future studies should therefore also investigate whether character traits or behavioural outcomes are more important for both social and non-social judgments, or how the two may interact.

The additional explorations proposed here could help to further validate the present findings, thus improving their generalisability.

## Conclusion

Our study presents preliminary evidence that episodic memory recall is more accurate for social compared to non-social behaviours and events. As there is currently limited research directly investigating differences for such effects in social and non-social contexts, our findings expand upon the existing episodic memory literature. These findings provide additional support to social theories of human development and emphasise the role of sociality within cognitive functions. We also found that memory recall was more accurate for events that were consistent with previously learnt target-trait associations, although this effect only remained significant in the non-social condition. Lastly, we observed a positivity bias for episodic memory recall in both social and non-social contexts, highlighting a need for further investigation into what factors may drive positivity biases in episodic memory. Understanding this could further inform approaches aiming to decrease negativity biases and increase positivity biases in cognition. We argue that the effects observed in the present study provide some insights into the mechanisms that allow us to successfully navigate the myriad novel situations we encounter in our daily lives.

## Supporting information

S1 Appendix (PDF)

S1 FigStudy procedure flow chart.A flow chart of the study procedure. 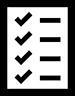. = self report measures (demographics, PHQ, O^2^S^3^, real-world social networks); 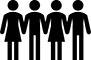 = episodic memory task (social condition); 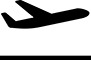 = episodic memory task (non-social condition).(TIFF)

S2 Fig Change in target ranking (post-task minus baseline) depending on target traits. Fig S2 depicts change in target ranking (post-task minus baseline) depending on target traits. S2a depicts changes in the non-social condition. S2b depicts changes in the social condition. From left to right for each condition for the different trait types (positive (non-social: organised, social: nice) vs negative (non-social: disorganised, social: mean)), the picture depicts individual data points showing each participants’ change in ranking (with jittering used to improve data point visibility), followed by a boxplot with the mean change in ranking across participants indicated by the bar, and lastly a frequency distribution of participants’ total change in ranking.
(TIFF)

S1 Table Episodic memory task means and SDs. Trait recall = number of correctly recalled target-trait associations in phase 2 (out of 24 associations per condition); Trait prediction = number of outcomes predicted (phase 4) based on learnt trait associations (out of 48 trials for each condition); Accuracy non-social = accuracy during the test phase (phase 5) for non-social episodic memory task condition (out of 48 trials for each condition); Accuracy social = accuracy during the test phase for social episodic memory task condition (out of 48 trials for each condition); Consistent = number of accurately recalled outcomes during the test phase that were consistent with prior knowledge (out of 24 trials for each condition); Inconsistent = number of accurately recalled outcomes during the test phase that were inconsistent with prior knowledge (out of 24 trials for each condition); Positive valence = number of accurately recalled outcomes during the test phase that were positive (airports were functioning well/people did something kind; out of 24 trials for each condition); Negative valence = number of accurately recalled outcomes during the test phase that were negative (airports were not functioning well/people did something mean; out of 24 trials for each condition).
(PDF)

S2 Table Correlations of predictor and outcome variables. * *p* < .05, ** *p* < .01, *** *p* < .001. Depressive symptoms = total score on the PHQ; Social sensitivity = total score on the O^2^S^3^; Size = sum of total number of in person and online friends; Quality = sum of total number of friends participants could ask a favour of and would trust to keep a secret; Satisfaction = sum of reported happiness with how often participants spend time with friends online and in person; Support = how supported participants felt by their friends; Accuracy non = accuracy for non-social episodic memory task condition; Accuracy social = accuracy for social episodic memory task condition.
(PDF)

S3 Table Summary of H2b analyses. Bolded text indicates statistically significant effects.
(PDF)

S4 Table Summary of exploratory analyses for effect of consistency on task accuracy (H2b) in the social compared to the non-social condition. Bolded text indicates statistically significant effects.
(PDF)

S5 Table Summary of contrast models for H2b analyses. Bolded text indicates statistically significant effects. Non indicates non-social condition; Social indicates social condition.
(PDF)

S6 Table Summary of exploratory trait memory effects on H1 and H2. Bolded text indicates statistically significant effects.
(PDF)

S7 Table Summary of H3b analyses. Bolded text indicates statistically significant effects.
(PDF)

S8 Table Summary of exploratory analyses for effect of valence on task accuracy (H3b) in the social compared to the non-social condition. Bolded text indicates statistically significant effects.
(PDF)

S9 Table Summary of contrast models for H3b analyses. Bolded text indicates statistically significant effects. Non indicates non-social condition; Social indicates social condition.
(PDF)

S10 Table Summary of H4 analyses. Bolded text indicates statistically significant effects. Size = sum of total number of in person and online friends; Quality = sum of total number of friends participants could ask a favour of and would trust to keep a secret; Satisfaction = sum of reported happiness with how often participants spend time with friends online and in person; Support = how supported participants felt by their friends.
(PDF)

S11 Table Effects of depressive symptoms on H1 and H3. Bolded text indicates statistically significant effects. Depressive symptoms indicate levels of depressive symptoms on the Patient Health Questionnaire (none = 0–4; mild = 5–9; high = 10+).
(PDF)

S12 Table Effects of social sensitivity on H1 and H3. Bolded text indicates statistically significant effects.
(PDF)
