# Supplementary material for: Episodic memory differences in social and non-social contexts
Source: PLoS One. 2026 Apr 2;21(4):e0342919. doi: 10.1371/journal.pone.0342919 (PMC13046140; doi:10.1371/journal.pone.0342919)
Supplement: S1 Appendix — (PDF) [file pone.0342919.s001.pdf]

Traits List

Social Condition

Positive Traits

Considerate

Charming

Sociable

Nice

Warm

Pleasant

Gentle

Generous

Caring

Sweet

Supportive

Thoughtful

Negative Traits

Bitter

Mean

Unfriendly

Cruel

Cold

Harsh

Spiteful

Unpleasant

Unkind

Awful

Angry

Evil

Non-Social Condition

Positive Traits

Clean

Neat

Safe

Efficient

Reliable

Organised

Productive

Orderly

Dependable

Quick

Sanitary

Tidy

Negative Traits

Uncertain

Untidy

Messy

Crowded

Ineffective

Unreliable

Cluttered

Dirty

Slow

Disorderly

Useless

Unclean

Behavioural Sentences List

Social Condition

Positive Behaviours

X offered to help Y paint their house

X helped Y find their lost dog

X visited Y when they were sick in the hospital

X gave Y their coat when it was cold

X talked to Y at the company picnic for an hour

X comforted Y when they were having a hard week

X lent money to Y when they were in a financial crisis

X helped Y pack their luggage for a work trip

X went grocery shopping for Y when they were sick

X shared their umbrella with Y in a rainstorm

X volunteered several hours a week to help Y when they first arrived in the country

X stayed up late to help Y with a project

X took Y out to lunch on their birthday

X offered to help Y fix a fence

X picked up Y so they could go see the parade

X always remembers Y's birthday

X helped Y fix their roof

X paid for Y's dinner

X helped push Y's car out of a snow bank

X volunteered to stay late to help Y at work

X smiled and greeted Y when they ran into each other

X tried to involve Y, who is very shy, in social activities

X allowed Y to stay with them temporarily when Y was evicted

X had a welcoming party for Y when they started at the new job

X invited Y to go have lunch with their friends

X gave Y lifts when they were stranded

X took Y out for a drink to celebrate their promotion

X gave Y a ride to their high school reunion

X spent time one summer teaching Y to play baseball when they were younger

X complimented Y on their clothing and appearance

X always laughs at Y’s jokes, even when they’re not funny

X gave Y a house-warming present when they first moved in

X recommended Y for a new job opening at their company

X recommended a good plumber for Y when their taps weren’t working

X bought Y concert tickets that they wanted

X baked Y some cookies

X helped Y fix their garage door

X went to an important doctor’s appointment with Y to help ease their anxiety

X lent Y their phone when theirs lost battery

X helped Y complete their work

X bought Y medicine when they were feeling ill

X gave Y feedback for an upcoming presentation

X lent Y their car when theirs broke down

X offered to walk Y’s dog when they were injured

X complimented Y’s new haircut

X offered to help Y run errands

X lent Y their lawnmower after Y’s broke down

X bought Y a souvenir on their last holiday

Negative Behaviours

X refused to let Y join them for lunch

X was irritated when Y asked to borrow their phone

X yelled at Y because they were late

X drove through a puddle, purposely splashing Y

X let their dog go to the bathroom on Y’s yard

X sniggered when Y asked a question during a work meeting

X ignored Y’s request to borrow some tools

X refused to help Y with a work assignment

X laughed at Y when they tripped and fell

X got into a fight with Y during a work dinner

X started an argument with Y in front of their home

X refused to help Y fix their report

X shoved Y when walking past them

X closed the elevator door before Y could get on

X ignored Y in the office for several weeks

X refused to loan notes to Y when they were ill

X swore at Y when they made an error

X stole into a parking place while Y was backing in

X slammed the door to the office in Y's face

X ridiculed Y behind their back

X scratched Y's car with their keys

X put sugar in Y's gas tank

X hit Y's car and left the scene of the accident

X ignored Y at a drinks function

X started a false rumour about Y

X broke Y's album and neither apologised nor replaced it

X continually berated Y in front of their co-workers

X refused to hold the door for Y when they were injured

X turned in Y's project under their own name

X stole money from Y

X ridiculed Y in front of their friends

X ignored all of Y's calls

X interrupted and spoke over Y at a group lunch

X filed a baseless complaint about Y at work

X wrote a rude comment under Y's social media post

X dropped their rubbish in front of Y's house when taking a walk

X did not return Y's favourite book and didn't apologise

X didn't inform Y of an upcoming project when they were off sick

X didn't respond to Y's call for help when they were stranded at the bus stop

X insulted Y's hair in public

X threw out Y's lunch after seeing it in the office fridge

X left mean notes on Y's desk when they were new to the company

X pretended they didn't recognise Y when they saw each other in public

X gave Y a fake address for the company dinner

X hid Y's car keys when they had to stay late to finish work

X refused to take on Y's ideas during a work meeting

X always criticises Y's work, even when they have done nothing wrong

X always makes fun of Y's sports teams

Non-Social Condition

Positive Behaviours

X is now running flights to Y following a recent expansion of its flight routes

X is now running flights to Y after hiring more ground staff

X is running all flights to Y on time after settling scheduling issues

X is running flights to Y following a remodel of one of its terminals

X is operating flights to Y after solving a system malfunction

X is running flights to Y due to good management

X is running flights to Y after updating its safety protocol

X is running flights to Y after updating its sanitary infrastructure throughout the airport

X is operating flights to Y after reorganising the baggage handling system

X is running flights to Y after quickly resolving an issue with baggage transfers

X is now operating flights to Y after minimising operational costs

X is running flights to Y after updating their cybersecurity measures

X is now running flights to Y again after quickly resolving a mechanical issue

X is running flights to Y after updating their operating system to the newest version

X is running flights to Y because of a well-managed booking system

X is running flights to Y because of a new air traffic control systems

X is running flights to Y because of good communication between staff members

X is running flights to Y due to perfectly maintained airlock doors

X is running flights to Y after resolving staff disputes

X is now running flights to Y having fixed baggage loading issues

X is running flights to Y after resolving communication errors between refuelling staff

X is running flights to Y after renovating some of the departure gates

X is running flights to Y after resetting all computers and fixing technical errors

X is operating flights to Y after refurbishing the interior of the airport

X is running flights to Y after fixing all connection issues between control centres

X is running flights to Y after organising extra staff to cover for mishaps

X is running flights to Y after improving its flight radar system

X is now running flights to Y after settling a dispute with its catering company

X is running flights to Y after replacing its old luggage scales

X is running flights to Y after organising a backup pilot in case of emergencies

X is running flights to Y after organising an effective plan to resolve common issues

X is running flights to Y after conducting checks on electrical systems

X is running flights to Y after conducting checks on plumbing systems

X is running flights to Y after organising prompt clean-up after previous flights

X is running flights to Y after proper password communication between staff

X is running flights to Y after ensuring all cabin crew arrive on site at least 2 hours prior to departures

X is running flights to Y after implementing a new system for workers to confirm their shift

X is running flights to Y after ordering extra safety equipment for emergencies

X is running flights to Y after ensuring all planes were at optimal flying capacity

X is running flights to Y after implementing a new flight departure schedule

X is running flights to Y after ensuring all air conditioning systems within the planes were in good working order

X is running flights to Y after improving communications between cabin and ground crews

X is running flights to Y after promptly resolving a safety hazard on the runway

X is running flights to Y after utilising a new policy that protected its staff

X is running flights to Y after updating their management system

X is now running flights to Y after promptly fixing an issue with airplane GPS systems

X is now running flights to Y after quickly resolving an issue with the airplane wings

X is running flights to Y after effective communication with catering companies

Negative Behaviours

X has stopped flights to Y to investigate luggage delays

X has delayed all flights to Y due to miscommunication between staff

X has cancelled all flights to Y to investigate a security threat

X has stopped flights to Y due to air traffic restrictions

X has stopped flights to Y due to a fuel shortage

X has cancelled all flights to Y due to poor management of departure times

X has delayed all flights to Y to inspect a hazardous runway

X has cancelled flights to Y due to malfunctioning airport security scanners

X has cancelled flights to Y because of an electrical wiring issue

X has delayed all flights to Y due to issues with the baggage removal processes

X has delayed flights to Y due to a shortage of food for the flights

X has cancelled flights to Y due to clashes with other flight times

X has delayed flights to Y due to a system error

X has delayed flights to Y due to a mechanical error with the plane's landing systems
X has cancelled all flights to Y after a reduction in its landing spaces
X has delayed all flights to Y due to all aircrafts going offline for programming maintenance
X has cancelled flights to Y due to a major computer glitch
X has delayed flights to Y because of a disease outbreak
X has delayed flights to Y due to a plumbing issue flooding the runway
X has delayed flights to Y due to a control tower failure needing urgent fixing
X has cancelled flights to Y because of an error with the booking system
X has cancelled flights to Y due to faulty airlock doors that were left unattended during maintenance
X has delayed flights to Y due to miscommunication with other airports regarding flight times
X has delayed flights to Y due to a lack of ground staff.
X has delayed flights to Y due to late cabin crew arrivals
X has delayed flights to Y due to an electricity failure within cabins, leaving them with no power
X has cancelled flights to Y due to a miscalculation of the baggage loads
X has cancelled flights to Y due to contamination of their water supply
X has delayed flights to Y due to a broken gangway
X has delayed flights to Y due to a delay in transferring connecting baggage
X has cancelled flights to Y because of delays between flights
X has delayed flights to Y due to a miscommunication with refuelling staff
X has delayed flights to Y because of unsanitary cabin conditions requiring prompt clean-up
X has delayed flights to Y due to malfunctioning runway lighting
X has delayed flights to Y due to malfunctioning speaker systems
X has cancelled flights to Y due to a lockdown of computer systems
X has delayed flights to Y due to unsafe passenger boarding conditions
X has cancelled flights to Y due to miscalculation of safety equipment
X has delayed flights to Y due to an incomplete safety protocol
X has delayed flights to Y due to a management issue
X has cancelled flights to Y after miscommunicating with passengers about boarding requirements
X has delayed flights to Y due to cabin crew strike
X has delayed flights to Y due to issues with the GPS systems

X has delayed flights to Y because of technical issues with the satellite systems

X has delayed flights to Y due to a pest infestation

X has cancelled flights to Y after a dispute between pilots

X has delayed flights to Y because of life jacket shortages

X has delayed flights to Y due to traffic on the runway
