# Supplementary material for: Episodic memory differences in social and non-social contexts
Source: PLoS One. 2026 Apr 2;21(4):e0342919. doi: 10.1371/journal.pone.0342919 (PMC13046140; doi:10.1371/journal.pone.0342919)
Supplement: S1 Table — Trait recall = number of correctly recalled target-trait associations in phase 2 (out of 24 associations per condition); Trait prediction = number of outcomes predicted (phase 4) based on learnt trait associations (out of 48 trials for each condition); Accuracy non-social = accuracy during the test phase (phase 5) for non-social episodic memory task condition (out of 48 trials for each condition); Accuracy social = accuracy during the test phase for social episodic memory task condition (out of 48 trials for each condition); Consistent = number of accurately recalled outcomes during the test phase that were consistent with prior knowledge (out of 24 trials for each condition); Inconsistent = number of accurately recalled outcomes during the test phase that were inconsistent with prior knowledge (out of 24 trials for each condition); Positive valence = number of accurately recalled outcomes during the test phase that were positive (airports were functioning well/people did something kind; out of 24 trials for each condition); Negative valence = number of accurately recalled outcomes during the test phase that were negative (airports were not functioning well/people did something mean; out of 24 trials for each condition). (PDF) [file pone.0342919.s004.pdf]

S1 Table. Episodic memory task means and SDs.

	Mean (SD)
**Trait recall**	16.02 (6.41)
Non-social	14.62 (6.07)
Social	17.41 (6.45)
**Trait prediction**	31.13 (7.77)
Non-social	28.13 (6.29)
Social	34.13 (7.97)
**Accuracy non-social**	28.77 (4.55)
Consistent	15.06 (2.84)
Inconsistent	13.71 (3.22)
Positive valence	15.58 (3.29)
Negative valence	13.19 (3.01)
**Accuracy social**	36.24 (5.43)
Consistent	18.24 (3.08)
Inconsistent	18.00 (3.23)
Positive valence	18.72 (3.17)
Negative valence	17.52 (3.62)

Trait recall = number of correctly recalled target-trait associations in Phase-2 (out of 24 associations per condition); Trait prediction = number of outcomes predicted (Phase-4) based on learned trait associations (out of 48 trials for each condition); Accuracy non-social = accuracy during the test phase (Phase-5) for non-social episodic memory task condition (out of 48 trials for each condition); Accuracy social = accuracy during the test phase for social episodic memory task condition (out of 48 trials for each condition); Consistent = number of accurately recalled outcomes during the test phase that were consistent with prior knowledge (out of 24 trials for each condition); Inconsistent = number of accurately recalled outcomes during the test phase that were inconsistent with prior knowledge (out of 24 trials for each condition); Positive valence = number of accurately recalled outcomes during the test phase that were positive (airports were functioning well/people did something kind; out of 24 trials for each condition); Negative valence = number of accurately recalled outcomes during the test phase that were negative (airports were not functioning well/people did something mean; out of 24 trials for each condition).
