# Supplementary material for: Episodic memory differences in social and non-social contexts
Source: PLoS One. 2026 Apr 2;21(4):e0342919. doi: 10.1371/journal.pone.0342919 (PMC13046140; doi:10.1371/journal.pone.0342919)
Supplement: S2 Table — * p < .05, ** p < .01, *** p < .001. Depressive symptoms = total score on the PHQ; Social sensitivity = total score on the O2S3; Size = sum of total number of in person and online friends; Quality = sum of total number of friends participants could ask a favour of and would trust to keep a secret; Satisfaction = sum of reported happiness with how often participants spend time with friends online and in person; Support = how supported participants felt by their friends; Accuracy non = accuracy for non-social episodic memory task condition; Accuracy social = accuracy for social episodic memory task condition. (PDF) [file pone.0342919.s005.pdf]

S2 Table. Correlations of predictor and outcome variables.

Variable	1	2	3	4	5	6	7	8
1. Age								
2. Depressive symptoms	−.29**							
3. Social sensitivity	−.22**	.63**						
4. Size	−.04	−.10	−.16*					
5. Quality	−.03	−.03	−.13	.58**				
6. Satisfaction	.01	−.24**	−.30**	.06	.10			
7. Support	.05	−.24**	−.38**	.26**	.29**	.58**		
8. Accuracy non	.04	.07	.07	−.13	−.10	−.11	−.06	
9. Accuracy social	−.00	.12	.14*	−.03	−.01	−.05	−.10	.37**

* $p < .05$, ** $p < .01$, *** $p < .001$. Depressive symptoms = total score on the PHQ; Social sensitivity = total score on the O^2S^3; Size = sum of total number of in person and online friends; Quality = sum of total number of friends participants could ask a favour of and would trust to keep a secret; Satisfaction = sum of reported happiness with how often participants spend time with friends online and in person; Support = how supported participants felt by their friends; Accuracy non = accuracy for non-social episodic memory task condition; Accuracy social = accuracy for social episodic memory task condition.
