# Supplementary material for: Episodic memory differences in social and non-social contexts
Source: PLoS One. 2026 Apr 2;21(4):e0342919. doi: 10.1371/journal.pone.0342919 (PMC13046140; doi:10.1371/journal.pone.0342919)
Supplement: S3 Table — Bolded text indicates statistically significant effects. (PDF) [file pone.0342919.s006.pdf]

S3 Table. Summary of H2b analyses.

	Accuracy			
Predictors	*df*	*F*	*p*	R^2m/R^2c
				0.28/0.47
Condition	**642.00**	**153.27**	**<.001**	
Consistency	**642.00**	**27.74**	**<.001**	
Condition x Consistency	**642.00**	**9.28**	**.002**	

Bolded text indicates statistically significant effects.
