# Supplementary material for: Episodic memory differences in social and non-social contexts
Source: PLoS One. 2026 Apr 2;21(4):e0342919. doi: 10.1371/journal.pone.0342919 (PMC13046140; doi:10.1371/journal.pone.0342919)
Supplement: S4 Table — Bolded text indicates statistically significant effects. (PDF) [file pone.0342919.s007.pdf]

S4 Table. Summary of exploratory analyses for effect of consistency on task accuracy (H2b) in the social compared to the non-social condition.

	**Accuracy**							
Predictors	*df*	*F*	*p*	R^2m/R^2c	*df*	*F*	*p*	R^2m/R^2c
	**Social Condition**				**Non-social Condition**			
				0.00/0.48				0.05/0.16
Consistency	214.00	1.26	.264		**214.00**	**24.24**	**<.001**	

Bolded text indicates statistically significant effects.
