# Supplementary material for: Episodic memory differences in social and non-social contexts
Source: PLoS One. 2026 Apr 2;21(4):e0342919. doi: 10.1371/journal.pone.0342919 (PMC13046140; doi:10.1371/journal.pone.0342919)
Supplement: S5 Table — Bolded text indicates statistically significant effects. Non indicates non-social condition; Social indicates social condition. (PDF) [file pone.0342919.s008.pdf]

S5 Table. Summary of contrast models for H2b analyses.

	**Accuracy**			
Predictors	*β*	*95% CI*	*t*	*p*
Non (Consistent vs Inconsistent)	**0.37**	**0.20 - 0.55**	**5.27**	**<.001**
Social (Consistent vs Inconsistent)	0.07	−0.11 - 0.24	0.96	.808
Non vs Social (Consistent)	**−0.87**	**−1.05 - −0.70**	**−12.38**	**<.001**
Non vs Social (Inconsistent)	**−1.18**	**−1.35 - −1.00**	**−16.69**	**<.001**

Bolded text indicates statistically significant effects. Non indicates non-social condition; Social indicates social condition.
