# Supplementary material for: Episodic memory differences in social and non-social contexts
Source: PLoS One. 2026 Apr 2;21(4):e0342919. doi: 10.1371/journal.pone.0342919 (PMC13046140; doi:10.1371/journal.pone.0342919)
Supplement: S6 Table — Bolded text indicates statistically significant effects. (PDF) [file pone.0342919.s009.pdf]

S6 Table. Summary of exploratory trait memory effects on H1 and H2.

	Accuracy			
Predictors	*df*	*F*	*p*	R^2m/R^2c
A: *Effects of trait memory on H1 Analysis*				
				0.39/0.57
Condition	**231.18**	**295.46**	**<.001**	
Trait recall	**416.58**	**21.94**	**<.001**	
B: *Effects of trait memory on H2a Analysis*				
				0.10/0.17
Consistency	**643.38**	**12.43**	**<.001**	
Trait recall	**498.81**	**76.03**	**<.001**	
C: *Effects of trait memory on H2b Analysis*				
				0.30/0.46
Condition	**668.35**	**124.74**	**<.001**	
Consistency	**641.30**	**27.47**	**<.001**	
Trait recall	**583.65**	**21.12**	**<.001**	
Condition x Consistency	**641.30**	**9.19**	**.003**	

Bolded text indicates statistically significant effects.
