# Supplementary material for: Episodic memory differences in social and non-social contexts
Source: PLoS One. 2026 Apr 2;21(4):e0342919. doi: 10.1371/journal.pone.0342919 (PMC13046140; doi:10.1371/journal.pone.0342919)
Supplement: S7 Table — Bolded text indicates statistically significant effects. (PDF) [file pone.0342919.s010.pdf]

S7 Table. Summary of H3b analyses.

	Accuracy			
Predictors	*df*	*F*	*p*	R^2m/R^2c
				0.29/0.43
Condition	**642.00**	**234.07**	**<.001**	
Valence	**642.00**	**71.47**	**<.001**	
Condition x Valence	**642.00**	**8.97**	**.003**	

Bolded text indicates statistically significant effects.
