# Supplementary material for: Episodic memory differences in social and non-social contexts
Source: PLoS One. 2026 Apr 2;21(4):e0342919. doi: 10.1371/journal.pone.0342919 (PMC13046140; doi:10.1371/journal.pone.0342919)
Supplement: S9 Table — Bolded text indicates statistically significant effects. Non indicates non-social condition; Social indicates social condition. (PDF) [file pone.0342919.s012.pdf]

S9 Table. Summary of contrast models for H3b analyses.

	Accuracy			
Predictors	*β*	*95% CI*	*t*	*p*
Non (Negative vs Positive)	**−0.62**	**−0.80 - −0.43**	**−8.45**	**<.001**
Social (Negative vs Positive)	**−0.31**	**−0.49 - −0.13**	**−4.22**	**<.001**
Non vs Social (Negative)	**−1.12**	**−1.30 - −0.93**	**−15.30**	**<.001**
Non vs Social (Positive)	**−0.81**	**−0.99 - −0.63**	**−11.06**	**<.001**

Bolded text indicates statistically significant effects. Non indicates non-social condition; Social indicates social condition.
