# Supplementary material for: Episodic memory differences in social and non-social contexts
Source: PLoS One. 2026 Apr 2;21(4):e0342919. doi: 10.1371/journal.pone.0342919 (PMC13046140; doi:10.1371/journal.pone.0342919)
Supplement: S10 Table — Bolded text indicates statistically significant effects. Size = sum of total number of in person and online friends; Quality = sum of total number of friends participants could ask a favour of and would trust to keep a secret; Satisfaction = sum of reported happiness with how often participants spend time with friends online and in person; Support = how supported participants felt by their friends. (PDF) [file pone.0342919.s013.pdf]

S10 Table. Summary of H4 analyses.

	**Accuracy**			
Predictors	*df*	*F*	*p*	R^2m/R^2c
***A:** Effects of personal network size*				
				0.36/0.59
Condition	**212.00**	**160.03**	**<.001**	
Size	373.99	2.95	.086	
Condition x Size	212.00	1.34	.248	
***B:** Effects of personal network quality*				
				0.36/0.59
Condition	212.00	160.66	**<.001**	
Quality	374.22	1.89	.170	
Condition x Quality	212.00	1.12	.291	
***C:** Effects of personal network satisfaction*				
				0.36/0.59
Condition	213.00	25.28	**<.001**	
Satisfaction	376.82	2.30	.130	
Condition x Satisfaction	213.00	0.42	.519	
***D:** Effects of personal network support*				
				0.36/0.59
Condition	213.00	54.29	**<.001**	
Support	376.80	0.57	.452	
Condition x Support	213.00	0.62	.431	

Bolded text indicates statistically significant effects. Size = sum of total number of in person and online friends; Quality = sum of total number of friends participants could ask a favour of and would trust to keep a secret; Satisfaction = sum of reported happiness with how often participants spend time with friends online and in person; Support = how supported participants felt by their friends.
