# Supplementary material for: Episodic memory differences in social and non-social contexts
Source: PLoS One. 2026 Apr 2;21(4):e0342919. doi: 10.1371/journal.pone.0342919 (PMC13046140; doi:10.1371/journal.pone.0342919)
Supplement: S11 Table — Bolded text indicates statistically significant effects. Depressive symptoms indicate levels of depressive symptoms on the Patient Health Questionnaire (none = 0–4; mild = 5–9; high = 10+). (PDF) [file pone.0342919.s014.pdf]

S11 Table. Effects of depressive symptoms on H1 and H3.

	Accuracy			
Predictors	*df*	*F*	*p*	R^2m/R^2c
***A:** Effects of depressive symptoms on H1*				
				0.36/0.58
Condition	**204.00**	**191.57**	**<.001**	
Depressive symptoms	365.43	0.16	.687	
Condition x Depressive symptoms	204.00	0.47	.493	
***B:** Effects of depressive symptoms on H3a*				
				0.06/0.11
Valence	**616.00**	**20.64**	**<.001**	
Depressive symptoms	551.57	0.01	.934	
Valence x Depressive symptoms	616.00	1.44	.230	
***C:** Effects of depressive symptoms on H3b*				
				0.29/0.42
Condition	**612.00**	**109.55**	**<.001**	
Valence	**612.00**	**25.60**	**<.001**	
Depressive symptoms	744.80	0.77	.382	
Condition x Valence	612.00	2.38	.124	
Condition x Depressive symptoms	612.00	1.58	.209	
Valence x Depressive symptoms	612.00	3.39	.066	
Condition x Valence x Depressive symptoms	612.00	1.26	.263	

Bolded text indicates statistically significant effects. Depressive symptoms indicate levels of depressive symptoms on the Patient Health Questionnaire (none = 0-4; mild = 5-9; high = 10+).
