# Supplementary material for: Episodic memory differences in social and non-social contexts
Source: PLoS One. 2026 Apr 2;21(4):e0342919. doi: 10.1371/journal.pone.0342919 (PMC13046140; doi:10.1371/journal.pone.0342919)
Supplement: S12 Table — Bolded text indicates statistically significant effects. (PDF) [file pone.0342919.s015.pdf]

S12 Table. Effects of social sensitivity on H1 and H3.

	**Accuracy**			
Predictors	*df*	*F*	*p*	R^2m/R^2c
A: *Effects of social sensitivity on H1*				
				0.37/0.59
Condition	**200.00**	**67.20**	**<.001**	
Social sensitivity	356.42	0.74	.391	
Condition x Social sensitivity	200.00	1.44	.232	
B: *Effects of social sensitivity on H3a*				
				0.06/ 0.11
Valence	604.00	1.99	.159	
Social sensitivity	541.85	0.02	.897	
Valence x Social sensitivity	**604.00**	**5.15**	**.024**	
C: *Effects of social sensitivity on H3b*				
				0.30/0.43
Condition	**600.00**	**50.11**	**<.001**	
Valence	**600.00**	**7.75**	**.006**	
Social sensitivity	727.04	0.05	.828	
Condition x Valence	**600.00**	**4.74**	**.030**	
Condition x Social sensitivity	600.00	0.03	.865	
Valence x Social sensitivity	600.00	1.85	.174	
Condition x Valence x Social sensitivity	600.00	0.82	.365	

Bolded text indicates statistically significant effects.
